# Researching gender and loneliness differently

**DOI:** 10.1111/nyas.15283

**Published:** 2025-01-06

**Authors:** Manuela Barreto, David Matthew Doyle, Marlies Maes

**Affiliations:** ^1^ Department of Psychology University of Exeter Exeter UK; ^2^ VUmc Amsterdam UMC Amsterdam The Netherlands; ^3^ Social and Behavioural Sciences Utrecht University Utrecht The Netherlands

**Keywords:** gender, gender stereotypes, intersex, loneliness, nonconformity, social stigma, transgender

## Abstract

The majority of research on loneliness considers gender by comparing the loneliness reported by men and women. Drawing on current conceptualizations of gender and its effects, we propose alternative ways in which gender should be examined in relation to loneliness. To do so, we consider multiple gender‐related factors and the role of the social environment, particularly societal ideologies about what gender is and how it should be expressed. We provide examples of how this expanded conceptualization can contribute to an improved understanding of loneliness by focusing on the impact of gender nonconformity, gendered life experiences, and couple relationships. We highlight the need for more research and evidence to fill existing gaps in understanding. We conclude that the field can move forward by considering the role of biological sex, gender identity, gender expression, gender roles, gender relational experiences, and sexual orientation, as well as the social norms against which these are experienced. To truly examine the role of gender in loneliness, we need to consider the normative context where some, but not others, are minoritized and marginalized, as well as move beyond binary notions of gender to include those with nonbinary, transgender, and intersex identities.

## Graphical abstract

Loneliness research should go beyond simplistic approaches to gender that ignore social context and restrict gender to the male/female binary. Instead, the impact of gender on loneliness needs to consider biological sex, gender identity, gender expression, gender role endorsement, gendered relational experiences, and sexual orientation, and consider them against a social environment characterized by specific gender representations and social norms.

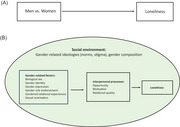

## INTRODUCTION

Loneliness can be defined as a painful feeling that arises when someone perceives a deficiency in the quantity or quality of their social relationships.^1^ This is different from social isolation, which is a more objective state of physical separation from others. Such feelings of loneliness, especially when prolonged, detrimentally affect mental health (e.g., increased depression^2^ and anxiety^3^) and physical health (e.g., more sleep problems,^4^ cardiovascular disease,^5^ and a higher risk of early mortality).^6^ In addition to being problematic for individuals, loneliness has been estimated to have high costs for societies, in part through increased use of health systems and reduced productivity.^7^, ^8^, ^9^, ^10^ The high prevalence and far‐reaching consequences of loneliness have led scholars^11^ and policymakers^12^, ^13^ to call for loneliness to be treated as a public health priority. In response, some governments have developed strategies to address loneliness and social isolation, and promote social connection.^14^ To do so effectively, it is important to understand who tends to experience loneliness and why. In this context, gender is one of the variables that needs to be considered, but exactly which gender group experiences loneliness more often, and why, is not entirely clear.

We argue this lack of clarity stems from the limited way in which gender has been examined in this area of research. Indeed, research on loneliness has examined the link between gender and loneliness mainly by comparing men's and women's scores on loneliness measures. Yet, current conceptualizations of gender have moved beyond this simplistic binary framing to consider not only nonbinary gender identities, but also the interplay between identity and context, raising the need to update how gender is examined in this area of research. In this paper, we start by providing an overview of how gender has been examined in relation to loneliness and subsequently outline how else we think this can be done. We then provide some examples of how this expanded conceptualization can contribute to an improved understanding of loneliness while, at the same time, pointing out the need for more research and evidence to fill existing gaps in understanding.

## HOW GENDER HAS BEEN CONSIDERED IN THE LONELINESS LITERATURE

**FIGURE 1:**
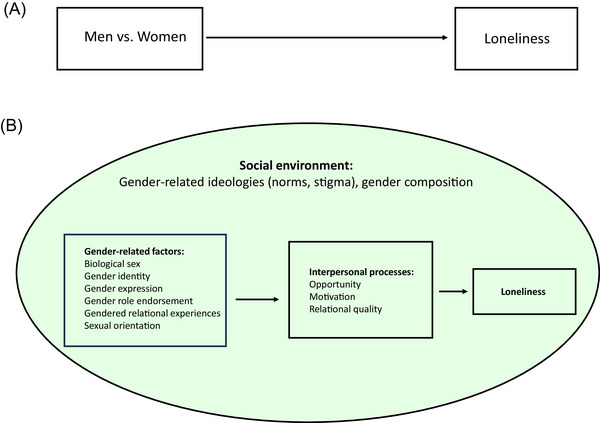
(A) Traditional perspective on how gender is considered in the loneliness literature. (B) Alternative perspective on how to conceptualize gender and loneliness by considering how gender‐related factors shape specific interpersonal processes within a social environment.

The link between gender and loneliness has most often been approached by comparing men's and women's experiences with loneliness (Figure 1A). Regarding what men and women mean by loneliness, qualitative studies indicate that men and women define loneliness similarly.^15^ With regard to mean scores on measures of loneliness, studies vary in whether they report more loneliness in men, in women, or no difference at all. In an attempt to systematize existing knowledge, meta‐analyses have shown that differences between men and women are overall close to zero (*g* = 0.07) when the measure does not directly ask whether participants feel lonely.^16^ When participants are directly asked how lonely they felt, a small difference is found (*g* = −0.23), indicating higher loneliness among women than among men.^15^ This finding is consistent with the prevailing hypothesis that men and women experience similar levels of loneliness, but men are less likely to admit to feeling lonely because they experience more stigma associated with loneliness.^17^ However, the literature examining gender differences in the stigma surrounding loneliness does not support the idea that men necessarily experience more stigma associated with loneliness than do women.^18^, ^19^

Although there do not seem to be large, meaningful differences in mean loneliness levels between men and women, there could still be differences in the factors that predict loneliness for each of these groups. A review on gender differences in predictors of loneliness found that relationship status—most often theorized and examined by reference to heterosexual relationships—was more strongly associated with loneliness for men than women, but for most other predictors, the evidence for gender differences was lacking or inconclusive.^15^ Moreover, several studies examined associations of loneliness separately for men and women, without testing whether the magnitude of these associations actually differed for these two gender groups. In sum, current evidence for how gender impacts loneliness is limited and unclear.

In addition, understandings of how gender might impact loneliness are also theoretically underdeveloped. Indeed, although researchers have examined differences between men and women's loneliness scores, scholars have not provided much theoretical rationale for why men and women might, overall, differ in their levels of loneliness. Some studies have found that men and women differ, for example, regarding time spent with family and friends,^20^, ^21^ social network size,^22^ and the importance of dyadic versus group interactions.^23^ These findings have often led to the assumption that men and women might differ in how often they experience loneliness. However, this assumption is flawed because loneliness is not necessarily related to time spent with others, social network size, or the importance afforded to social relationships of various types. Rather, loneliness arises when one perceives a deficit in the quantity or quality of one's social relationships.^1^ In other words, loneliness does not necessarily arise when one's network is small, but only when it is smaller than one would like it to be. Similarly, even with many friends, or when married, one can feel lonely if one perceives one's relationship quality as unsatisfying. Strikingly, when studies provide a theoretical rationale for gender differences in loneliness, they usually refer to how gender might affect actual or desired social relationships, not to the *discrepancy* between these.

## HOW ELSE WE SHOULD THINK ABOUT GENDER

Figure 1B displays an alternative approach that takes into account multiple aspects of gender as well as the social context in which both gender and loneliness are experienced. While most past work on gender and loneliness merely compared men and women (Figure 1A), we argue that to understand how gender affects loneliness, we need to consider other aspects of gender such as biological sex, gender identities, gender expression, gender roles, gender relational experiences, and sexual orientation, and how these combine to affect people's social relationships.

Work using the traditional binary view of gender often additionally uses the term gender interchangeably with, or as a proxy for, biological sex or sex assigned at birth. Therefore, much of what we know, or more aptly do not know, about gender differences in loneliness centers around the experiences of people who identify as cisgender women compared to people who identify as cisgender men. However, gender is much broader than this simplistic framing allows. Scholars have described gender as a multidimensional construct,^24^ with the term “gender bundle”^25^ proposed to refer to distinct aspects of gender, including sex/gender assigned at birth, current gender identity, gender roles and stereotypes, and gender expression. Notably, some of these aspects of gender are relatively more internal (e.g., gender identity), while others are more external (e.g., sex assigned at birth). Crucially, all are influenced by social feedback processes through which identities are or are not affirmed, negotiated, and changed,^26^ with discrepancies across more internal and external aspects of identity often emerging and shaping how gender plays out in social interactions.^27^ By taking this on board, we can account for, and understand the experiences of, intersex, transgender, and other gender‐diverse individuals. We, therefore, suggest that this more nuanced portrayal could help expand current understandings of how gender is (and is not) related to loneliness. Indeed, gender minorities (such as intersex and transgender individuals) have been found to experience particularly high levels of loneliness,^28^, ^29^ but these findings are currently relegated to niche areas of research and disregarded by mainstream conceptualizations of loneliness and how it is affected by gender.

In addition to expanding our approach to what constitutes gender, it seems crucial to consider gender in context, or the social environment that colors and affords meaning and value to gendered life experiences (see Figure 1B). As has been theorized before,^30^ to understand the implications and impact of any gender identity requires a consideration of context, both in terms of its gender composition and of the prevalent social norms against which gender is defined and expressed. For example, as with other social groups, men and women are likely to experience more loneliness in settings where they are under‐represented, marginalized, or devalued.^29^, ^31^ As such, even if we focus only on comparing cisgender women to cisgender men, it might be wise to consider, for example, whether the context in which their loneliness is assessed is one in which they are minoritized. This indicates that it is fairly meaningless to compare, for example, men and women in general, without specifying the gender norms that surround them and how these interact with the way in which they live their gender identities.

Acknowledging the importance of gender roles and stereotypes, which not only influence identity content but also how people of different genders behave, starts opening up new avenues for the consideration of how gender affects social relationships and potentially loneliness. For example, we become able to examine how traditional gender‐related ideologies can drive loneliness among sexual and gender minorities by involving expectations about heterosexuality and cis‐normativity that marginalize members of these groups.^32^, ^33^ This consideration also directs us to attend to intersections between gender and sexual orientation as well as culture, age, and disability, all of which are associated with specific stereotypes that differentially impact the social relationships of individuals of different genders.

In the next sections, we provide some examples of how considering gender in this way can stimulate progress in understandings of how loneliness is experienced by all.

## (NON‐)CONFORMITY AND SOCIAL EXCLUSION

Both the social environment and broader societal attitudes play important roles in shaping one's social opportunities and willingness to engage with others, with those whose identities are marginalized more likely to be left out.^31^ Gendered societal expectations influence individual loneliness by dictating who is liked, validated, and welcomed, and who is not. As conceptualized by stigma and ambivalent sexism frameworks, gender‐related ideologies are expressed in stereotypes, prejudice, and discrimination that perpetuate (cisgender) male dominance by encouraging compliance and punishing nonconformity.^34^, ^35^ These societal attitudes and beliefs are still dominated by binary and cis/heteronormative beliefs, which in turn are associated with restricted ideals about how individuals should be, behave, and live their lives.^36^ One is expected to identify with the sex one has been assigned at birth and endorse the corresponding gender roles, including taking part in heterosexual relationships, choosing a gender‐appropriate level of work‐life balance, and ideally producing offspring that perpetuate this status quo. Those who do not conform to these ideals are often derogated and excluded from social interactions, with likely implications for their feelings of loneliness.^29^ Unsurprisingly, then, more loneliness is reported by those who do not identify as cisgender male or female, as well as by those in same‐sex relationships.^37^

One of the routes through which gender nonconformity might increase loneliness is exposure to invalidating views about one's gender. For example, transgender individuals often report that others fail to use their chosen name and pronouns, sometimes deliberately.^38^ Intersex individuals often contend with others’ lack of understanding of their sex/gender, which is unintelligible to others precisely because it does not fit simple normative understandings of what gender is—which makes them feel lonely.^39^, ^40^ This lack of identity validation and affirmation can elicit low self‐worth and reduce self‐concept clarity,^41^ both of which can increase the likelihood of loneliness.^42^, ^43^ Moreover, lack of gender identity validation can limit identity expression, reducing feelings of authenticity, which plays an important role in intimacy and belonging.^44^ In addition, those who do not endorse traditional binary gender identities are often targeted by prejudiced attitudes and behaviors, which can directly reduce their opportunities for satisfying social interactions,^29^ as well as lead to communication apprehension and loneliness.^45^

Irrespective of their gender identity, individuals might or not conform to traditional gender roles in how they choose to live their lives. Interestingly, the evidence suggests that gender roles can promote loneliness both in those who endorse them and in those who do not. For example, an increase in women's share of household and caring tasks during the COVID‐19 pandemic was associated with an increase in loneliness among women (but not among men).^46^ For men, endorsement of stereotypical beliefs about masculinity (such as that the man, rather than the woman, should be the breadwinner) was positively associated with loneliness, and less conservative gender attitudes protected against loneliness, particularly among older men.^47^ The precise mechanisms underlying these effects are as yet unknown, but it is possible that they are associated with the stress implied by the restrictive expectations associated with gender norms, irrespective of whether they lead to disadvantage (e.g., for women) or to advantage (e.g., for men).

Evidence that deviating from gender roles is associated with loneliness can be found, for example, for women who pursue careers in stereotypically male domains or positions. Indeed, research has documented that it is “lonely at the top,” especially for women leaders.^48^ This likely happens because women leaders suffer backlash due to stereotype violation,^49^ which can isolate them from satisfying social networks. Although some women in these environments have access to women's networks that protect them from loneliness,^50^ others cope with perceived pervasive gender‐based discrimination by distancing themselves from other women.^51^ Evidence additionally suggests that often women who work in male‐dominated environments feel that they need to present themselves and behave in ways that limit their feelings of authenticity,^48^ which are an important precursor of satisfying social interactions.

In sum, nonconformity with socially prescribed gender identities, stereotypes, and roles is an important precursor of loneliness that is obscured if we merely compare the loneliness reported by men and women.

## LONELY GENDERED LIFE EXPERIENCES

Another way in which gender affects loneliness is by influencing life experiences that can be associated with loneliness. While it has been pointed out in the loneliness literature that life transitions can influence loneliness,^52^ insufficient attention has as yet been dedicated to understanding how gendered life transitions can be, or to uncovering the social consequences of life experiences that are gendered.

Research on the impact of parenthood on loneliness reveals mixed findings,^53^ with studies showing that some parents experience considerable loneliness, but failing to specify what circumstances might make parents lonely. We suggest that this issue would be better understood if more attention were dedicated to the sociocultural and gendered aspects of parenthood and of the social support needed by, and not always available to, mothers and fathers (of different genders and sexual orientations) in different cultural contexts. For example, in most societies, women are traditionally expected to thrive in pregnancy and motherhood, for which they are supposedly evolutionarily suited—even though this expectation clashes with many women's experiences.^54^ Because of these expectations, women are often left to fend for themselves when they become new mothers, which is an experience that is associated with loneliness.^55^ Fathers, on the other hand, are traditionally expected to continue working after starting a family. Fathers who choose to, instead, stay at home taking care of their family often experience stigma associated with this counterstereotypical choice, an experience that is associated with feelings of depression, isolation, and disconnection.^56^ While this might, in some contexts, lead to similar levels of loneliness for men and women, these experiences are very different and so are the needs associated with them. In addition, it remains to be understood how these gendered experiences are reflected in the social lives of those who do not endorse traditional gender roles, such as gender minorities or those in same‐sex relationships.

Informal caregiving is also a common life experience that has been linked to loneliness^57^ with insufficient attention dedicated to its gendered aspects. Worldwide, informal caregiving is more common among women than men.^58^ However, one study found that the impact of informal caregiving on loneliness was greater for men than for women,^59^ potentially because it is counternormative for men. Another study revealed that for grandmothers caring for a grandchild was more protective of loneliness if done regularly and intensively, whereas for grandfathers caring for a grandchild was more protective if it was only occasional.^60^ Understanding the effects of gender on loneliness, therefore, requires closer attention to not only what experiences are most frequent for each gender group, but also how they are perceived and supported in society.

Some life experiences are tied to elements of biological sex, like menstruation and menopause, and tend to be gendered. There is a growing understanding that these experiences are shaped by a number of biological but also psychological and social factors. Research has begun to uncover, for example, that loneliness is associated with the severity of premenstrual symptoms in adolescent girls,^61^ and that endometriosis can be experienced as isolating and lonely.^62^ These associations might emerge not only because the physical pain associated with these conditions is likely to impair participation in social activities, but also because these conditions are still widely stigmatized, motivating those who menstruate to conceal their experience,^63^ which can impair social relationships.^44^ In addition to facing these obstacles, nonbinary and transmasculine individuals who menstruate need to contend with the possibility that menstruation will cause gender dysphoria and/or invalidate their gender identity in others’ eyes, enhancing their motivation to conceal menstruation and the isolation elicited by doing so.^64^

There is also some suggestive evidence that menopause can be associated with loneliness.^65^ Those who experience menopause undergo physical and psychological changes associated with the drop in endogenous estrogen production that range from (potentially disfiguring) skin problems to heightened anxiety, depression, and lowered self‐confidence.^66^ Menopause symptoms can motivate women to withdraw from social activities. In addition, many women feel a lack of understanding for what they are going through in menopause, in part because their (cisgender male) relational partners are unaware of what menopause entails, or what support to provide.^67^ There is also a large degree of misunderstanding regarding the ways in which menopause can be experienced and felt by transmasculine and nonbinary people, including within healthcare services.^68^ The lack of attention dedicated to the link between menopause and social relationships means that it is as yet unclear exactly how and when it is linked to loneliness. For example, menopause occurs along with other life transitions, such as the “empty nest syndrome” caused by children leaving the parental home, widowhood, or divorce, and more research is needed to understand specific social needs at these life stages and how they can be met. In addition, menopause emerges due to hormonal changes and is often accompanied by hormone replacement therapy, but the social impact of these hormonal fluctuations is rarely researched. Similarly, the potential implications of gender‐affirming hormone therapy, during medical gender transition, for social health and psychosocial functioning,^69^ are as yet under‐researched.

In sum, gender affects loneliness by shaping life experiences that can promote loneliness, while at the same time, similar life experiences can impact loneliness differently for different genders depending on the beliefs dominant in a given society.

## LONELINESS IN COUPLES

Emotional loneliness is conceptualized as the loneliness one experiences as a result of feeling a lack of emotional intimacy, either because one does not have a romantic partner or because this relationship lacks empathy or intimacy.^70^ It is, therefore, unsurprising that being in a relationship versus single generally exhibits a stronger protective effect against emotional but not social loneliness, and this appears to be more the case for heterosexual men compared to women.^71^ One possible reason for this gender difference in the buffering effect of relationship status against loneliness is that—in heterosexual relationships—men are more likely to derive fulfillment of intimacy needs from a romantic partner compared to women.^72^, ^73^ Rather than simply an innate difference, this is likely to be shaped to a large extent by gender roles and expectations within heterosexual relationships.^74^, ^75^ For example, women tend to do more emotion work within heterosexual relationships (e.g., initiating emotional conversations, offering encouragement and support) compared to men,^74^ which contributes to building intimacy and potentially decreasing loneliness in couples. Other research suggests that even when both partners do emotion work, it is more effective in increasing relationship satisfaction when done by women.^76^ In a striking example, there is evidence that male romantic partners are six times more likely to separate after their female partners are diagnosed with a serious physical illness compared to the reverse,^77^ again suggesting that a caring role is more normative for women in heterosexual relationships, but the implications of these differences for those who contradict stereotypes (e.g., men who care for their ill partners) remain underexamined.

Importantly, not every romantic relationship is protective against loneliness, nor are all single people lonely. Those in relationships of low quality or relationships marked by conflict appear to be particularly vulnerable to loneliness (even more so than those who are single), and there is some evidence that this effect of relationship quality may not differ based on gender.^78^ Associations between singlehood and loneliness may be driven in part by a perceived failure to meet (potentially gendered) societal norms and expectations that people will settle down, marry, and have kids.^79^ Of course, the sociohistorical context shapes how singlehood is perceived across different cultures and life stages,^80^, ^81^ as well as by gender.^82^ However, despite prominent stereotypes of unhappy single women (e.g., “the crazy cat lady,” “the lonely spinster”), some recent research reveals that single women may experience lower desire for a partner as well as greater relationship status satisfaction and general well‐being compared to single men.

Research on loneliness in sexual minority couples, or those involving gender minorities, is scarce. Relationship status does similarly seem to be an important predictor of loneliness for sexual minorities, with those in romantic relationships reporting less loneliness compared to those who are not,^83^, ^84^ although this beneficial effect may not apply to asexuals.^85^ For those in romantic relationships, there is evidence that same‐sex couples tend to share work in relationships—including emotion work—more equally than heterosexual couples.^75^ This once again points to the fact that differences in emotion work may be based at least in part on normative gender roles, as same‐sex couples appear to adapt gendered cultural scripts to build intimacy in a manner suited to either masculine (e.g., emotional autonomy and independence) or feminine (e.g., intensive emotion work and desire for intimacy) social norms.^75^ Similarly, same‐sex parents may divide childcare tasks more evenly than heterosexual couples, which has been linked to greater relationship satisfaction and better adjustment of children,^86^ which in turn could buffer against loneliness. However, same‐sex couples face unique challenges that heterosexual couples do not, with implications for relationship quality and loneliness. For example, social stigma at internalized, interpersonal, and structural levels can damage romantic relationships and increase loneliness among sexual minorities.^87^ Thus, restrictive norms around gender and sexual orientation have consequences for those whose family formation is perceived as non‐normative within a given society.

Irrespective of sexual orientation, another factor that relates to social isolation and loneliness is gender violence, including but not restricted to intimate partner violence and sexual abuse. It is commonly acknowledged that isolation from friends and family is a key mechanism through which gender violence is maintained. In fact, studies show that isolation is sometimes in itself a key form of marital abuse,^88^ and those who are isolated in this way cannot rely on their partner to fulfill their needs for social connection. What is less understood is how this plays differently for male and female victims and how it is linked to notions of femininity and masculinity underlining victim and perpetrator roles. For example, while it is understood that male perpetrators are often motivated by particular ideals of masculinity, it is less acknowledged how these interfere with male victim's ability to identify and report their victimization. Moreover, although gender violence more commonly targets female victims, the experiences of male victims are often neglected, an example of what has been labeled “ethical loneliness.”^89^ In addition, although it is clear that transgender and gender‐diverse people are frequently exposed to intimate partner violence,^90^ how this might be linked to loneliness is as yet underexamined.

In sum, gender and sexual orientation affect how people behave within romantic relationships, as well as how they are regarded by others, which in turn is likely to have implications for the loneliness experienced within (and outside of) couples.

## THE IMPORTANCE OF CONTEXT

The pervasiveness of restrictive gender‐related beliefs and the extent to which they are supported by other societal beliefs and structures vary across institutions and regions. This contextual variation provides a background against which individuals are perceived to endorse nontraditional gender identities or behaviors, or are excluded when they do so. A nonbinary person who was assigned female at birth and presents with relatively more feminine characteristics may be commonly identified as a woman in a society with traditionalist cultural values and gender norms. As a result, they may be expected to play a specific caregiving role within a family,^17^ which could lead to loneliness because they do not feel comfortable with this role and how it shapes their relationships.

One study suggests that the social and psychological causes and consequences of menopause are likely to be strongly related to societal‐level gendered attitudes toward aging, which marginalize older women.^91^ Research has also documented that loneliness is less prevalent in countries with higher levels of gender inequality, where there are provisions to protect the quality of marital relationships (such as no‐fault divorce laws).^92^ How this might interact with individual gender is, however, unclear. The absence of protective policies can impact individual loneliness by communicating identity devaluation and the presence of such policies can help individuals cope with the discrimination they encounter.^87^, ^93^ Indeed, research has found that perceived discrimination had a stronger impact on loneliness among sexual minorities in US states that had fewer (vs. more) policies protecting sexual minority rights.^87^ Research in this area is, however, still scarce, though promising in its clarification of how environments can be improved to prevent loneliness.

Those who do not conform to traditional gender roles are likely to feel particularly isolated in contexts where various sources of traditional beliefs about gender converge. For example, it is possible that traditional gender roles have a stronger effect on loneliness in cultures where norm adherence is more strictly expected (tight vs. loose cultures).^94^ Ideologies related to gender and sexual orientation have also been linked to religiosity (across different religions).^95^ Even though at the individual level religiosity can protect from loneliness,^96^ it is likely that environments dominated by a stronger adherence to religious beliefs are more likely to marginalize those who do not conform to traditional gender norms, increasing their loneliness.

This contextual nature of gender processes influencing loneliness can obscure gender differences, which might emerge only for some subgroups or within some contexts. Moreover, this contextual variation clarifies that loneliness is not inherent to any specific gender identity.^31^ Simply put, people are likely to be more lonely when (or where) their needs are neglected or devalued.

## CONCLUSIONS

In this paper, we argue for the need to research the link between gender and loneliness differently. While prior research in this area has found inconsistent effects of gender, meta‐analyses have indicated that gender differences in loneliness are negligible.^15^, ^16^ Importantly, whereas gender differences are not always very informative, the absence of such differences is not either, since it obscures important effects that might emerge for subgroups of men or women, or in specific contexts. To move the field forward, we argue that gender needs to be considered beyond simplistic notions of gender and the male versus female binary to encompass nonbinary, transgender, and intersex identities among others. We additionally highlight that to truly examine the role of gender in loneliness, we need to move beyond comparing people of different genders to consider the normative context where some, but not others, are minoritized and marginalized. Crucially, being marginalized is not a trait; it is a process that emerges from the relationship between an identity and the stigmatizing attitudes, behaviors, and structures that dominate some social environments. This idea helps us overcome the notion that some groups are more at risk of, or vulnerable to, loneliness by themselves, and highlights the role of exclusion in specific social environments, one that is also more amenable to change than essentialized individual traits. With this perspective, we also begin to see how gender‐related ideologies impact loneliness in other groups, such as sexual minorities. We acknowledge that some of the evidence in direct support of our argument is missing, with some studies, for example, focusing on variables that are only imperfectly related to loneliness, such as social isolation or relationship quality. This lack of evidence is, however, precisely likely to be due to the narrow way in which gender has been considered in this field. We, therefore, encourage researchers to broaden their scope to shed light on how gender affects loneliness for all individuals. In doing so, we will not only further the understanding of the link between gender and loneliness, but also extend existing insights into the social impacts of marginalization.

## AUTHOR CONTRIBUTIONS

All authors contributed to the conceptualization, writing, and editing of this manuscript.

## COMPETING INTERESTS

The authors have no competing interests to declare.

### PEER REVIEW

The peer review history for this article is available at: https://publons.com/publon/10.1111/nyas.15283.
